# The Effect of Polyethylene Glycol Addition on Wettability and Optical Properties of GO/TiO_2_ Thin Film

**DOI:** 10.3390/ma14164564

**Published:** 2021-08-13

**Authors:** Azliza Azani, Dewi Suriyani Che Halin, Kamrosni Abdul Razak, Mohd Mustafa Al Bakri Abdullah, Marcin Nabiałek, Muhammad Mahyiddin Ramli, Mohd Fairul Sharin Abdul Razak, Andrei Victor Sandu, Wojciech Sochacki, Tomasz Skrzypczak

**Affiliations:** 1 Geopolymer & Green Technology, Centre of Excellence (CEGeoGTech), Universiti Malaysia Perlis (UniMAP), Perlis 02600, Malaysia; aazliza@hotmail.com (A.A.); kamrosni@unimap.edu.my (K.A.R.); mustafa_albakri@unimap.edu.my (M.M.A.B.A.); mmahyiddin@unimap.edu.my (M.M.R.); fairulsharin@unimap.edu.my (M.F.S.A.R.); 2 Faculty of Chemical Engineering Technology, Universiti Malaysia Perlis (UniMAP), Perlis 02600, Malaysia; 3 Department of Physics, Częstochowa University of Technology, 42-200 Częstochowa, Poland; nmarcell@wp.pl; 4 Faculty of Electronic Engineering Technology, Universiti Malaysia Perlis (UniMAP), Perlis 02600, Malaysia; 5 Faculty of Materials Science and Engineering, Gheorghe Asachi Technical University of Iasi, 71 D. Mangeron Blv., 700050 Iasi, Romania; sav@tuiasi.ro; 6 Faculty of Mechanical Engineering and Computer Science, Częstochowa University of Technology, 42-200 Częstochowa, Poland; wojciech.sochacki@pcz.pl (W.S.); t.skrzypczak@imipkm.pcz.pl (T.S.)

**Keywords:** titanium dioxide, graphene oxide, thin film, sol-gel, wettability

## Abstract

Modification has been made to TiO_2_ thin film to improve the wettability and the absorption of light. The sol-gel spin coating method was successfully used to synthesize GO/TiO_2_ thin films using a titanium (IV) isopropoxide (TTIP) as a precursor. Different amounts of polyethylene glycol (PEG) (20 to 100 mg) were added into the parent sol solution to improve the optical properties and wettability of the GO/TiO_2_ thin film. The effect of different amounts of PEG was characterized using X-ray diffraction (XRD) for the phase composition, scanning electron microscopy (SEM) for microstructure observation, atomic force microscopy (AFM) for the surface topography, ultraviolet–visible spectrophotometry (UV-VIS) for the optical properties and wettability of the thin films by measuring the water contact angle. The XRD analysis showed the amorphous phase. The SEM and AFM images revealed that the particles were less agglomerated and surface roughness increases from 1.21 × 10^2^ to 2.63 × 10^2^ nm when the amount of PEG increased. The wettability analysis results show that the water contact angle of the thin film decreased to 27.52° with the increase of PEG to 80 mg which indicated that the thin film has hydrophilic properties. The optical properties also improved significantly, where the light absorbance wavelength became wider and the band gap was reduced from 3.31 to 2.82 eV with the presence of PEG.

## 1. Introduction

In the early 1970s, Honda-Fujishima was a pioneer researcher of titanium dioxide (TiO_2_), which is also known as a photocatalyst semiconductor [1,2]. TiO_2_ is the most outstanding photocatalyst with many applications in many areas, especially the environment area [3]. In order to degrade the environmental pollutants, TiO_2_ must have a good self-cleaning property, particularly its optical property. Therefore, TiO_2_ can be added as a photocatalyst to cementitious material, paints, tiles and glass materials. Furthermore, as a diamagnetic material, TiO_2_ has the advantage of integrating magnetic recovery and photocatalysis performance to enhance environmental protection [4]. Its economic factor with good stability, and high photocatalytic activity has made TiO_2_ a favourable chemical, and has gained the attention of researchers, where almost all studies of photocatalytic degradation of pollutants involve TiO_2_.

In the case of TiO_2_ thin films, two mechanisms responsible for the self-cleaning property are the hydrophilic mechanism in which the material absorbs sunlight, leading to the degradation of the pollutants and the hydrophobic mechanism which causes the water drops to roll off the surface, removing the contaminants from the surface [5]. First, sunlight produced UV photons will be absorbed by TiO_2_ and generate electron and electron-hole pairs. The electron-hole will then react with hydroxyl ions when the hole reaches the particle surface and adsorb surface water, forming an electrically neutral but highly reactive hydroxyl radical.

There are several disadvantages of TiO_2_ photocatalysts that affect the performance of TiO_2_. These disadvantages are due to a number of factors: the fast recombination rate of electron-hole pairs, where the high recombination rate of photogenerated electrons and holes that coexist in the TiO_2_ lead to a lower rate of the desired chemical transformation of the absorbed light energy [6,7]; a huge band gap can cause low photo response of TiO_2_ into wider wavelengths (>380 nm) or inefficient exploitation of the visible light gap energy [8]; and aggregation and agglomeration of TiO_2_ affect the photoactivity as well as light absorption [9,10]. Thus, further study of TiO_2_ has been discussed to overcome these problems.

Some modification was made to TiO_2_ to improve the absorption of light including doping with metals or non-metals, forming hetero-junctions between TiO_2_ and other low band gap semiconductors, and fabrication of graphene-based semiconductor nanocomposites [11]. However, fewer noble metals are being used due to expensive costs and some factors that limit the usage of noble metals in large-scale applications despite having many advantages. Therefore, graphene oxide (GO) is the best substitution for noble metal. This is due to its special properties, including large specific surface areas, flexible structure, excellent mobility of charge carriers at room temperature, and good electrical and thermal conductivities. In addition, GO was known to be an electron-donating material, which can improve catalyst efficiency. This donating material reduces the recombination rate of electron–hole pairs by increasing the charge-carrier mobility [12]. The hybridization of graphene with TiO_2_ has the impact of decreasing the band gap energy. It shifts the absorption threshold towards the visible light region and allows more photons to be utilised from solar energy. To further enhance the optical properties of the thin film, polyethylene glycol (PEG) was used to modify the surface of the thin film.

PEG prepared from a wide range of sizes and terminal functional groups is a linear synthetic polyether [13]. Increasing the active surface area due to porous structure and lowering the band gap of TiO_2_ are the advantages of this amazing additive, also known as a polymeric additive [14]. Furthermore, PEG is also a hydrophilic molecule, with extremely low fouling ability, cell adhesion and the ability to serve as a crosslinking agent, leading to a porous TiO_2_. These properties improve the binding of GO/TiO_2_ on the substrate [15].

This work aims to figure out the effect of TiO_2_ thin film with the addition of GO and PEG. This work also investigates the surface topography, optical properties and wettability of the produced GO/TiO_2_ thin film at the optimal amount of PEG addition. This modification is expected to improve the performance of the catalyst. It can improve the surface, expand the absorption into the visible spectrum and reduce the band gap energy. Furthermore, the thin films were synthesized and analyzed under the same conditions to obtain improved understanding of the processes.

## 2. Materials and Methods

### 2.1. Materials

Titanium (IV) isopropoxide 98% (TTIP) was used as a precursor with 15–20 sheets and 4% to 10% edge-oxidized graphene oxide powder (GO). Both were purchased from Sigma Aldrich, Saint Louis, MO, USA. Absolute ethanol used as a solvent was purchased from HMbG Chemicals Company. Acetic acid (99.5%) and Polyethylene Glycol 2000 (PEG) were purchased from Daejung Reagent Chemicals and Merck, respectively. All the chemicals purchased from several suppliers were of analytical reagent grades and were used as received, without further purifying. The sol-gel method was used with TTIP as a precursor to produce the thin film.

### 2.2. Preparations of GO/TiO_2_ Thin Film

First, to prepare the GO solution, 5 mg GO was dissolved in absolute ethanol and sonicated in an ultrasonic bath for 30 min. Next, Ti precursor sol was prepared by dissolving an amount of TTIP volume in 20 mL absolute ethanol and stirred continuously for 10 min. Then, the GO sol was mixed in Ti precursor sol for 10 min. This was followed by the addition of different amounts of PEG (20, 40, 60, 80 and 100 mg) and 0.10 mL of distilled water while stirring for another 10 min. An amount of 0.10 mL of acetic acid was added into the solution to function as a catalyst to the hydrolysis process. The solution was stirred vigorously for 1 h until a transparent sol was formed.

A clean glass substrate was used to deposit the thin film by the spin coating technique. A small amount of sol-gel solution was dropped onto the substrate and deposited through spin coating technique at 800 rpm for 30 s. Three layers of coating were deposited to ensure that the sol-gel entirely coated the substrate’s surface, followed by an annealing process at 350 °C for 1 h at a 10 °C/min annealing rate in the muffle furnace. The samples were annealed to improve the structural, surface topography and optical properties of the film [16]. The GO/TiO_2_ thin film with PEG were named 20 PEG, 40 PEG, 60 PEG, 80 PEG and 100 PEG.

### 2.3. Characterizations

The prepared thin films were characterized using X-ray diffraction (XRD) at 0°–70° with a Cu Kα (λ = 1.5046) at 40.0 kV, current of 30 mA and a scanning rate of 2° min^−1^. The XRD spectra were identified using the Hi-Score Plus Software. The microstructure of the thin films was analyzed using a scanning electron microscope (SEM). The films’ surface roughness and surface topography were examined using an atomic force microscope (AFM). The wettability of the thin films was measured by the contact angles of a drop of distilled water with the volume controlled on the surface, and the contact angle of the water drop on the films was first photographed with an optical microscope and then analyzed using ImageJ software. The optical absorption spectra were recorded using a UV-Vis spectrophotometer with a wavelength range of 350 to 600 nm. The calculation of the band gap energy for the thin films was performed using the Tauc equation:

(*αhv*) = A (*hv* − *Eg*)^1/2^ (1)

where, *h* = Planck constant (6.63 × 10^−34^ m^2^kg/s), *v* = photon frequency and *Eg* = band gap energy. The *Eg* value was obtained from the *(αhv)* graph at the y-axis and the photon energy at the x-axis. The value of *Eg* is given by *Eg = hv*, while (*αhv*)^2^ = 0 [17].

## 3. Results and Discussion

### 3.1. Phase Composition

**Figure 1:**
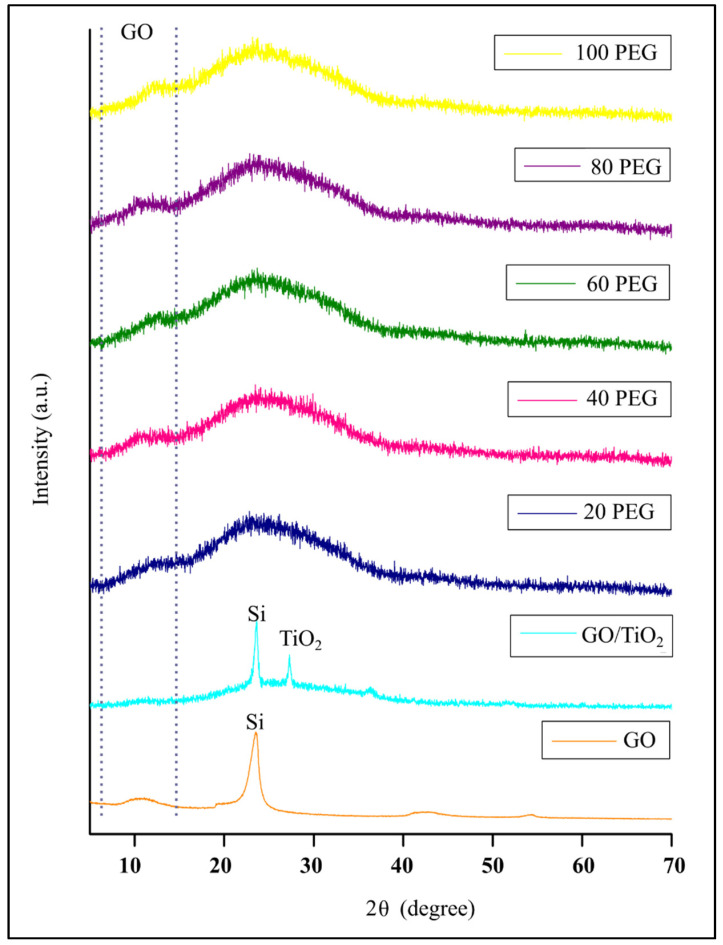
The XRD pattern of GO/TiO_2_ and GO/TiO_2_ with PEG thin films.

The GO/TiO_2_ thin film and GO/TiO_2_ with PEG thin film underwent X-ray Diffraction (XRD) analysis to determine and investigate the phase of the prepared thin film samples. The thin film samples were scanned in the range of 5° to 70° at the rate of 2° per minute. The XRD pattern of GO/TiO_2_, 20 PEG, 40 PEG, 60 PEG, 80 PEG and 100 PEG are presented in Figure 1. Based on the XRD pattern, a low intensity peak at 2θ = 10.3° was attributed to GO. However, when GO mixed with TiO_2_ at the 350 °C annealing temperature, mixed phases of anatase and brookite formed where 2θ = 24.8° can be attributed to the (101) plane of anatase while 2θ = 33.5° can be attributed to the (201) plane of brookite TiO_2_ (ICDD Card No. 01-070-8501). The GO peaks in GO/TiO_2_ thin film cannot be seen because the two peaks of TiO_2_ at 24.8° and 33.5° have a higher intensity than GO. When GO/TiO_2_ mixed with PEG at the 350 °C annealing temperature, the TiO_2_ crystal structure become amorphous, hence showed a broad feature between 20° to 30° with lower intensity. Then, the peak of GO in GO/TiO_2_ with PEG thin film showed up again at 2θ = 10.3°. With an increase in the amount of PEG, the GO peak was visible in the spectrum. This indicated the presence of PEG at the basal plane of GO. The single peak recorded at 2θ = 21.9° indicated the presence of silicon, resulting from the substrate.

The GO/TiO_2_ with PEG thin films revealed that all the samples form an amorphous TiO_2_ due to the interaction of GO/TiO_2_ and PEG and no peak patterns referred to the presence of anatase TiO_2_. In this situation, the heat energy supplied, which is equivalent to the quenching temperature used, is not enough to trigger the crystallization process [18]. The diffraction pattern does not show any anatase phase because of the annealing temperature factor whereby the anatase phase of TiO_2_ will slowly form from the brookite phase of the TiO_2_ when the annealing temperature increases to 500–600 °C [19]. The previous work done by Fischer et al. (2017) stated that the anatase phase of TiO_2_ would grow better at a higher temperature [20].

The diffraction pattern also showed that the addition of PEG does not improve the crystallinity of TiO_2_. The similar findings regarding PEG addition was obtained from previous work done by Nawawi et al. (2017) where the authors found that no phase transformation occurred since the thin films were prepared at low temperature. They also reported that phase transformation in the presence of PEG was observed when the annealing temperature was at 900 °C, while without PEG the phase transformation of TiO_2_ occurred at an annealing temperature of 700 °C [21]. However, the photodegradation activity of amorphous TiO_2_ was reported to be much better than the crystalline TiO_2_ [22]. The amorphous TiO_2_ showed a dominant effect on the surface of GO when there was a broad feature around 20–30°, which refers to the GO-TiO_2_ hybrid composite particles. In addition, a low intensity peak was found at 10.3° due to the separation of the GO sheets and less agglomeration of the deposition of TiO_2_ on the surface was found. Hence, this study showed that the TiO_2_ nanoparticles were homogeneously covered by GO at the surface of GO when there was a broad feature around 20–30°, which refers to the GO-TiO_2_ hybrid composite particles. In addition, a low intensity peak was found at 10.3° due to the separation of the GO sheets and less agglomeration of the deposition of TiO_2_ on the surface was found. Hence, this study showed that the TiO_2_ nanoparticles were homogeneously covered by GO [23].

### 3.2. Microstructure Analysis

**Figure 2:**
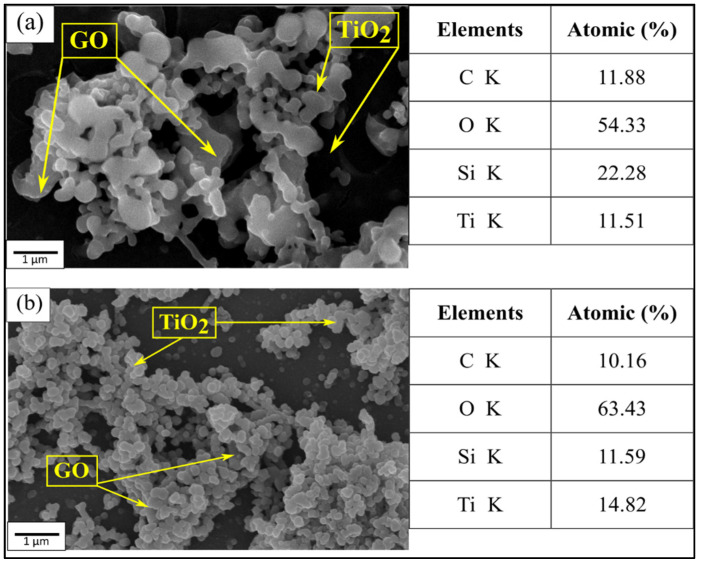
SEM and EXD analysis of (**a**) GO/TiO_2_ without PEG and (**b**) GO/TiO_2_ with PEG.

Figure 2 shows the microstructure and EDX analysis of GO/TiO_2_ and GO/TiO_2_ with PEG thin films which were observed under the scanning electron microscope (SEM) at 10,000× magnification. It can be seen that the particles of TiO_2_ were coated by GO and the existence of GO was proven in EDX analysis. The EDX analysis confirms the existence of carbon (C), oxygen (O) and titanium (Ti) generated from sol-gel composition while silica (Si) represents the element of the sample substrate. The signal for C mainly comes from the GO sheets, while those for Ti are from the TiO_2_ nanoparticles and O was contributed by TiO_2_ nanoparticles, and a small amount of oxygen-containing groups on GO sheets [24]. However, evaporation of PEG during the annealing process resulted in no detection of PEG in the EDX analysis.

From Figure 2a, it was observed that the molecules of the TiO_2_ with coated GO without the addition of PEG revealed an agglomeration having a large surface area, and the particle size of TiO_2_ became bigger. This condition is in agreement with a previous work in which the microstructure of TiO_2_ with GO addition showed that GO tightly covered the TiO_2_ surface and the covered TiO_2_ surface area increases with the presence of GO. Proper introduction of GO will both enhance the light absorption by having larger specific areas and the separation of photogenerated electrons and holes [25]. In Figure 2b, the reduction of agglomeration is clearly seen when GO/TiO_2_ is added with PEG, that prevents the agglomeration of nanoparticles [26].This observation is correlated with the XRD results discussed earlier whereby the intensity of the GO peak increased slightly due to the better dispersion of the GO/TiO_2_ surface. This result is well in agreement with Che Halin et al. (2020), in which they reported that agglomeration occurs when the combination of three set rate processes which involve consolidation and growth, wetting and nucleation and attrition and breakage [27]. However, the interaction between GO/TiO_2_ and PEG form an even film surface, porous structure and a crack-free film. In other previous work by Ramírez-Santos et al. (2012), the addition of PEG improved the cracking-free film and employed the porous structure of TiO_2_ with the PEG thin film [28]. The rough and porous surfaces obtained from SEM images are in line with AFM analysis where the surface roughness increases with increasing PEG concentration [29].

### 3.3. Surface Roughness and Topography Analysis

Atomic force microscopy (AFM) was used to investigate the surface roughness and surface topography of the GO/TiO_2_ thin film with the addition of 20, 40, 60, 80 and 100 mg of PEG. The surface roughness of the sample was evaluated from the Roughness Mean Square (RMS) values. Table 1 shows the RMS values of GO/TiO_2_, 20 PEG, 40 PEG, 60 PEG, 80 PEG and 100 PEG thin film’s surface roughness. The RMS values of GO/TiO_2_ decrease from 3.16 × 10^2^ to 1.21 × 10^2^ after adding 20 mg PEG, 1.53 × 10^2^ in 40 PEG, 1.60 × 10^2^ in 60 PEG and 2.63 × 10^2^ in 80 PEG. This is due to the oxidation and decomposition of PEG to carbon dioxide, which left numerous small cracks indicating, as mentioned before, pore-forming. It is in good agreement with the results obtained by Huang, Lei, Huang, Chen, and Chen, (2010) in their previous work where the templating reagent used to synthesize porous TiO_2_ thin film via the sol–gel method was PEG 2000. The presence of cracks formed on the surface resulted in forming nanopores [30]. However, the RMS value slightly decreased to 2.28 × 10^2^ in 100PEG. This is owing to the excess addition of PEG [31].

**Figure 3:**
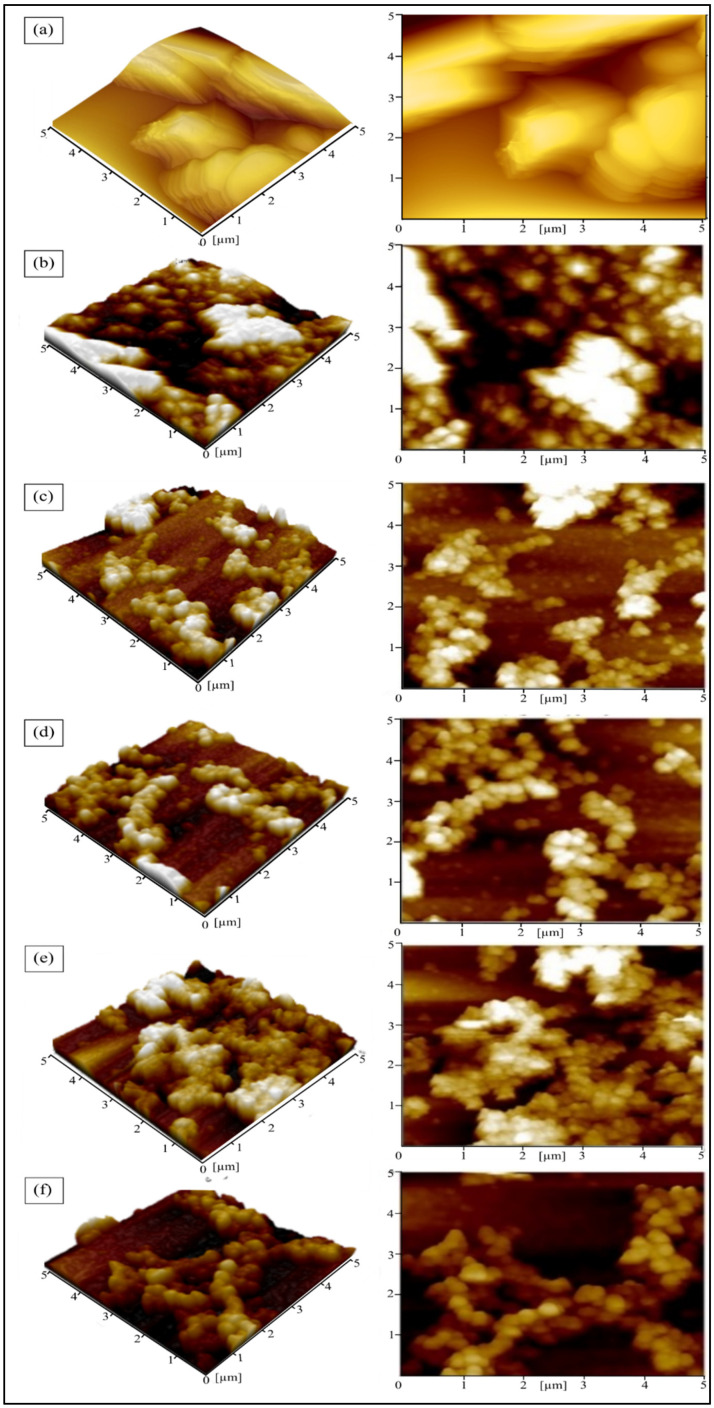
Surface topography of (**a**) GO/TiO_2_, (**b**) 20 PEG, (**c**) 40 PEG, (**d**) 60 PEG, (**e**) 80 PEG and (**f**) 100 PEG thin film.

The surface top view and three-dimensional view of GO/TiO_2_ and GO/TiO_2_ with PEG thin films are presented in Figure 3. From Figure 3a–f, the AFM images show the surface topography of the GO/TiO_2_ film with added PEG. It can be seen that the surface becomes smoother after the addition of PEG. However, when the amount of PEG was increased to 100 mg, the surface becomes rougher and the thickness becomes thicker as seen in Table 1. The result exhibits increasing surface roughness and uneven surface after the annealing process when the maximum height of the thin film increases from 427.03 to 893.67 nm as the amount of PEG increases. The roughness increase is connected with the formation of grown-up spherical size particles [32]. However, the addition of more PEG increased roughness. This can be due to the stress induced by the PEG removal from the film at an annealing temperature of 350 °C, as proven by a previous work done by Huang, Lei, Huang, Chen, and Chen, (2010). The higher the amount of PEG added to the sol solution, the more concentrated the resulting sol solution [30]. As the sol concentration increases, the resulting coating layer when the rotating coating process is performed, also thickens. Therefore, more solvent solution adheres to the substrate and makes a thicker thin film to be produced. The previous work done by Ramírez-Santos et al. (2012) also found that the TiO_2_ thin film layer added with PEG is thicker than the film layer which is not added with PEG [28].

### 3.4. Optical Properties

The optical analysis was conducted to determine the effect of adding PEG on GO/TiO_2_ and GO/TiO_2_ with PEG thin films including the absorption rate and band gap energy at 350 to 500 nm wavelength using an ultraviolet-visible spectrophotometer.

**Figure 4:**
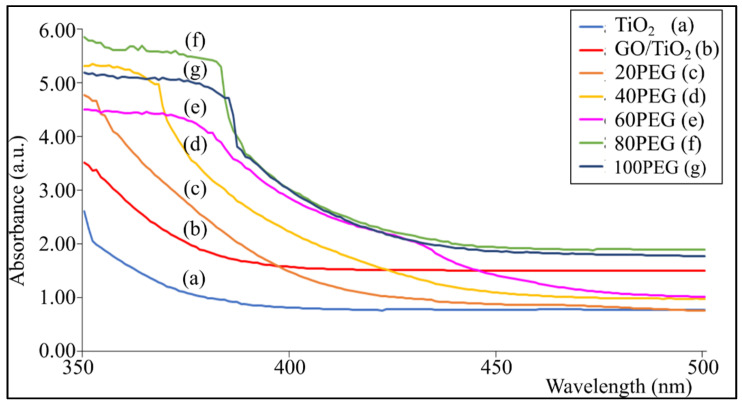
Absorbance versus wavelength of thin films with and without PEG.

Figure 4 shows the absorbance of GO/TiO_2_ and GO/TiO_2_ with PEG thin film at a wavelength range of 350–500 nm. The weights of additive added varied from 20 mg to 100 mg. The absorption shows an increasing trend as the amount of PEG increases. The absorption at a wavelength of 380 nm increases from 0.99 (a.u.) in bare TiO_2_ thin film followed by 1.85 (a.u.) in GO/TiO_2_, 2.44 (a.u.) in 20PEG, 3.26 (a.u.) in 40PEG, 4.08 (a.u.) in 60PEG and 5.44 (a.u.) in 80 PEG. The addition of PEG does have an important influence on the absorption of light with the increase of PEG used, and it can be seen that the absorption edge shifts within the absorption spectrum [33]. As shown in Figure 4, with the addition of PEG, there is a shift in the absorption spectra. With the addition of GO, the cut-off wavelength has been expanded into visible regions. Moreover, the cut-off wavelength of GO/TiO_2_ with PEG has shifted further than GO/TiO_2_ thin film.

As shown in Table 2, the cut-off wavelengths have shifted from 389 to 415 nm when GO is added and further shifted to 470 nm as more PEG is added. This indicates that the light absorption range of the thin film with the addition of PEG solution is wider than that of the film without PEG. Therefore, the light absorption rate can be attributed to the total surface area specific to the resulting thin film. However, the absorption at a wavelength of 380 nm, the cut-off wavelength and band gap energy were slightly reduced to 4.92 (a.u.), 460 nm and 2.86 eV, respectively in 100 PEG thin film due to excessive addition of PEG. This can also be seen in AFM analysis that shows the topography of the GO/TiO_2_ thin film surface is finer and more uniform when PEG is added and decreased with excess PEG. This gives a high amount of specific surface area and can absorb light better for photocatalytic activity to occur.

**Figure 5:**
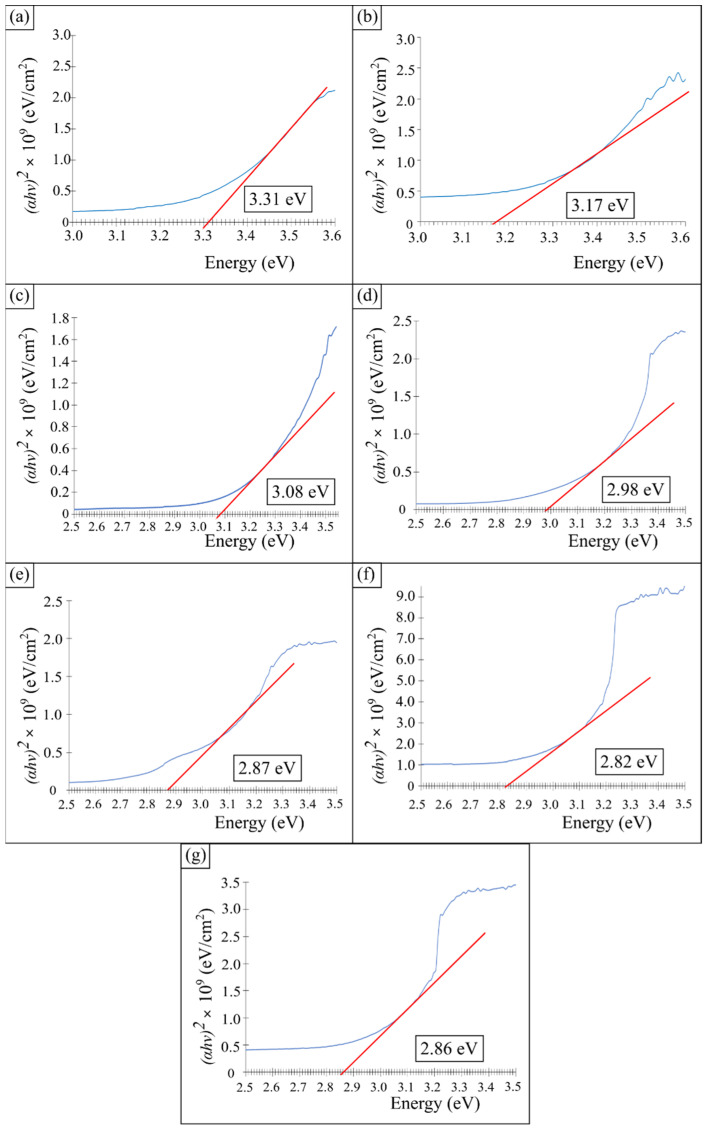
The band gap energy graph of (**a**) TiO_2_, (**b**) GO/TiO_2_, (**c**) 20 PEG, (**d**) 40 PEG, (**e**) 60 PEG, (**f**) 80 PEG and (**g**) 100 PEG thin film.

The calculation of the optical energy gap for the GO/TiO_2_ thin films with additives was also performed using the Tauc equation. Figure 5 shows the plot *(αhv)^2^* against photon energy *(hv)* to obtain the band gap energy value for the GO/TiO_2_ thin films with PEG. Based on the graph, the band gap energy value (Eg) is taken using the straight-line method on the x-axis. The value of the band gap energy obtained is tabulated in Table 2. The lowest band gap energy was obtained from 80 PEG, where the band gap energy value is 2.82 eV. As with the previous analysis of the cut-off wavelength, the band gap energy value also significantly improves with PEG.

These results prove that the addition of PEG can reduce the band gap energy that cannot be achieved by TiO_2_ thin film. From previous work, the value of the band gap energy is in the range of 3.20 to 3.56 eV [34]. However, a previous study by Gonçalves et al. (2018) revealed that the band gap energy measured for amorphous TiO_2_ is about 3.18 eV, which was slightly smaller than the value in the literature for crystalline anatase [35]. Therefore, the lower band gap energy was also contributed by the amorphous TiO_2_.

### 3.5. Wettability

**Figure 6:**
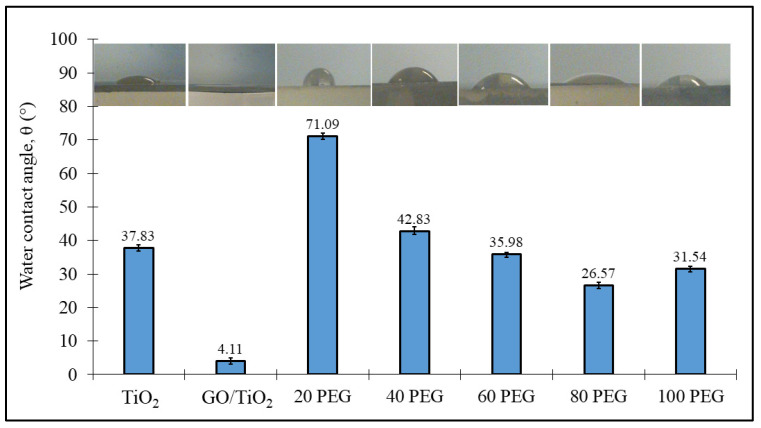
The water contact angle of GO/TiO_2_ thin films with different amounts of PEG.

Hydrophilic properties of GO/TiO_2_ and GO/TiO_2_ with PEG thin film were studied by measuring the water contact angles on the thin film. PEG was added to GO/TiO_2_ films to improve the performance of the photocatalyst as well as the self-cleaning activity. Figure 6 shows the water contact angles of a thin film with the addition of different PEG amounts. The results revealed that, generally, all the thin films with additives exhibit hydrophilic properties where all the angles measured were less than 90°. For example, according to Figure 5, the TiO_2_ thin film shows a water contact angle of 37.83°.

Interestingly, water contact angles of GO/TiO_2_ thin film drastically drops to 4.11° which means that the film is superhydrophilic (water contact angles ≤ 5°). This is because an anatase phase for GO/TiO_2_ thin film formed a better hydrophilic property. GO nanosheets are hydrophilic in nature because GO contains various hydrophilic functional groups [36]. However, the hydrophilic property of GO/TiO_2_ thin film decreases after the addition of PEG, but slowly increases when the amount of PEG increases. The greater the amount of PEG from 20, 40, 60, 80 and 100 mg, the greater the hydrophilic properties and decreased in water contact angle value, from 71.09° to 42.83°, 35.98° and 26.57°, respectively. This was due to the larger pore size produced from the thermal decomposition of PEG where the surface becomes rougher and slightly less hydrophilic. According to the previous work done by Yu et al. (2002) the surface roughness of TiO_2_ thin films were increased with an increasing amount of PEG in the precursor solution [37]. Moreover, further investigation found that the optimum amount of added PEG, 80 mg, increases the thin film’s hydrophilicity. The excess PEG addition slightly increased the water contact angle in 100 PEG to 31.54°.

From the results of the optical properties, thin films with additions of 80 mg PEG have the lowest band gap energy value, which is 2.82 eV—which provide high light absorption values by the films. Excessive light absorption can activate the film surface’s photocatalytic properties, which in turn contributes to the hydrophilic properties of the resulting film surface.

## 4. Conclusions

The GO/TiO_2_ thin film with different amounts of PEG was successfully synthesized from TTIP by using the sol-gel method. The sol-gel was deposited on the glass substrate by the spin coating technique at 800 rpm and underwent heat treatment at 350 °C. The thin films were characterized and it was found that the TiO_2_ is in an amorphous phase after the addition of PEG. The SEM images revealed that addition of PEG formed a reduced agglomeration of particles. The AFM images revealed that the RMS values of GO/TiO_2_ decrease from 3.16 × 10^2^ to 1.21 × 10^2^ after the addition of 20 mg PEG, and then increase from 1.21 × 10^2^ nm to 2.63 × 10^2^ nm when more PEG was added, due to pore formation during the stress induced by the PEG removal from the film during the annealing process. The wettability confirms that the GO/TiO_2_ thin film with the presence of PEG is hydrophilic when the water contact angle is less than 90° and the water contact angle of the thin film decreased from 71.09° with 20 mg PEG to 27.52° when PEG increased to 80 mg. The optical properties also improved, whereby the light absorption showed an increasing trend as the amount of PEG increased. The cut-off wavelength was further expanded from 389 to 415 nm into visible regions. Therefore, the band gap energy of the thin film was reduced from 3.17 to 2.82 eV because amorphous TiO_2_ was known to have an energy that was slightly smaller than the value for the crystalline anatase. This indicates that the PEG improves the optical properties of the thin film.

## Figures and Tables

**Table 1:** Roughness root mean square (RMS) and film thickness of GO/TiO_2_ and GO/TiO_2_ with different amounts of PEG thin films.

Samples	Film Thickness (nm)	Roughness Mean Square (RMS)
GO/TiO_2_	1740.32	3.16 × 10^2^
20 PEG	427.03	1.21 × 10^2^
40 PEG	638.42	1.53 × 10^2^
60 PEG	734.60	1.60 × 10^2^
80 PEG	893.67	2.63 × 10^2^
100 PEG	793.51	2.28 × 10^2^

**Table 2:** Band gap energy of GO/TiO_2_ thin film with various amount of PEG.

Samples	Cut-Off Wavelength (nm)	Band Gap Energy (E_g_)
TiO_2_	389	3.31
GO/TiO_2_	415	3.17
20 PEG	427	3.08
40 PEG	440	2.98
60 PEG	455	2.87
80 PEG	470	2.82
100 PEG	460	2.86

## Data Availability

The data presented in this study are available in this article.

## References

[1] Abdelamir A.I., Al-Bermany E., Hashim F.S. (2019). Enhance the Optical Properties of the Synthesis PEG/Graphene- Based Nanocomposite films using GO nanosheets. J. Phys. Conf. Ser..

[2] Bakardjieva S., Štengl V., Szatmary L., Subrt J., Lukac J., Murafa N., Nižňanský D., Cizek K., Jirkovsky J., Petrova N. (2006). Transformation of brookite-type TiO_2_ nanocrystals to rutile: Correlation between microstructure and photoactivity. J. Mater. Chem..

[3] Bakri A.S., Sahdan M.Z., Adriyanto F., Raship N.A., Said N.D.M., Abdullah S.A., Rahim M.S. (2017). Effect of annealing temperature of titanium dioxide thin films on structural and electrical properties. AIP Conf. Proc..

[4] Calderon-Moreno J., Preda S., Predoana L., Zaharescu M., Anastasescu M., Nicolescu M., Stoica M., Stroescu H., Gartner M., Buiu O. (2014). Effect of polyethylene glycol on porous transparent TiO_2_ films prepared by sol–gel method. Ceram. Int..

[5] Halin D.S.C., Razak K.A., Sukeri N.S.M., Azani A., Abdullah M.M.A.B., Salleh M.A.A.M., Mahmed N., Ramli M.M., Azhari A., Chobpattana V. (2020). The Effect of Polyethylene Glycol (PEG) on TiO_2_ Thin Films via Sol-Gel Method. IOP Conf. Ser. Mater. Sci. Eng..

[6] Demirci S., Dikici T., Yurddaskal M., Gültekin S., Toparli M., Celik E. (2016). Synthesis and characterization of Ag doped TiO_2_ heterojunction films and their photocatalytic performances. Appl. Surf. Sci..

[7] Dong H., Zeng G., Tang L., Fan C., Zhang C., He X., He Y. (2015). An overview on limitations of TiO_2_-based particles for photocatalytic degradation of organic pollutants and the corresponding countermeasures. Water Res..

[8] Fischer K., Gawel A., Rosen D., Krause M., Latif A.A., Griebel J., Prager A., Schulze A., Fischer K., Gawel A. (2017). Low-Temperature Synthesis of Anatase/Rutile/Brookite TiO_2_ Nanoparticles on a Polymer Membrane for Photocatalysis. Catalysts.

[9] Forbes P. (2008). Self-Cleaning Materials. Sci. Am..

[10] Gonçalves M.C., Pereira J.C., Matos J.C., Vasconcelos H.C. (2018). Photonic Band Gap and Bactericide Performance of Amorphous Sol-Gel Titania: An Alternative to Crystalline TiO_2_. Molecules.

[11] Guan K. (2005). Relationship between photocatalytic activity, hydrophilicity and self-cleaning effect of TiO_2_/SiO_2_ films. Surf. Coat. Technol..

[12] Halin D.S.C., Abdullah M.M.A.B., Mahmed N., Malek S.N.A.A., Vizureanu P., Azhari A.W. (2017). Synthesis and Characterization of TiO_2_/SiO_2_ Thin Film via Sol-Gel Method. IOP Conf. Ser. Mater. Sci. Eng..

[13] Halin D.S.C., Mahmed N., Salleh M.A.A.M., Sakeri A.M., Razak K.A. (2018). Synthesis and Characterization of Ag/TiO_2_ Thin Film via Sol-Gel Method. Solid State Phenom..

[14] Halin D.S.C., Razak K.A., Salleh M.A.A.M., Ramli M.I.I., Abdullah M.M.A.B., Azhari A.W., Nogita K., Yasuda H., Nabiałek M., Wysłocki J.J. (2021). Microstructure Evolution of Ag/TiO_2_ Thin Film. Magnetochemistry.

[15] Huang W., Lei M., Huang H., Chen J., Chen H. (2010). Effect of polyethylene glycol on hydrophilic TiO_2_ films: Porosity-driven superhydrophilicity. Surf. Coat. Technol..

[16] Karimi Z., Karimi L., Shokrollahi H. (2013). Nano-magnetic particles used in biomedicine: Core and coating materials. Mater. Sci. Eng. C.

[17] Low J., Cheng B., Yu J. (2017). Surface modification and enhanced photocatalytic CO_2_ reduction performance of TiO_2_: A review. Appl. Surf. Sci..

[18] Melcher J., Barth N., Schilde C., Kwade A., Bahnemann D.B.D. (2016). Influence of TiO_2_ agglomerate and aggregate sizes on photocatalytic activity. J. Mater. Sci..

[19] Moma J., Baloyi J. (2018). Modified Titanium Dioxide for Photocatalytic Modified Titanium Dioxide for Photocatalytic Applications.

[20] Nawawi W.I., Zaharudin R., Ishak M.A.M., Ismail K., Zuliahani A. (2016). The Preparation and Characterization of Immobilized TiO_2_/PEG by Using DSAT as a Support Binder. Appl. Sci..

[21] Ni Y., Wang W., Huang W., Lu C., Xu Z. (2014). Graphene strongly wrapped TiO_2_ for high-reactive photocatalyst: A new sight for significant application of graphene. J. Colloid Interface Sci..

[22] Nia M.H., Rezaei-tavirani M., Nikoofar A.R., Masoumi H., Nasr R., Hasanzadeh H., Jadidi M., Shadnush M. (2015). Stabilizing and Dispersing Methods of TiO_2_ Nanoparticles in Biological Studies. J. Paramed. Sci..

[23] Nurhamizah A., Zulkifli M., Juoi J.M. (2016). Effect of Additives on the Characteristic of Ag-TiO_2_ Coating Deposited on Specially Made Unglazed Ceramic Tile. Key Eng. Mater..

[24] Pellegrino F., Pellutiè L., Sordello F., Minero C., Ortel E., Hodoroaba V.-D., Maurino V. (2017). Influence of agglomeration and aggregation on the photocatalytic activity of TiO_2_ nanoparticles. Appl. Catal. B Environ..

[25] Ramírez-Santos A., Acevedo-Peña P., Córdoba E.M. (2012). Enhanced photocatalytic activity of TiO_2_ films by modification with polyethylene glycol. Quim. Nova.

[26] Šegota S., Ćurković L., Ljubas D., Svetličić V., Houra I.F., Tomašić N. (2011). Synthesis, characterization and photocatalytic properties of sol–gel TiO_2_ films. Ceram. Int..

[27] Saleh A.F., Jaffar A.M., Samoom N.A., Mahmmod M.W. (2017). Iraqi Ministry of Sciences and Technology. Effect Adding PVA Polymer on Structural and Optical Properties of TiO_2_ Thin Films. J. Al-Nahrain Univ. Sci..

[28] Sangchay W. (2016). The Self-cleaning and Photocatalytic Properties of TiO_2_ Doped with SnO_2_ Thin Films Preparation by Sol-gel Method. Energy Procedia.

[29] Shan A.Y., Ghazi T.I.M., Rashid S.A. (2010). Immobilisation of titanium dioxide onto supporting materials in heterogeneous photocatalysis: A review. Appl. Catal. A Gen..

[30] Sun S., Song P., Cui J., Liang S. (2019). Amorphous TiO_2_ nanostructures: Synthesis, fundamental properties and photocatalytic applications. Catal. Sci. Technol..

[31] Taherniya A., Raoufi D. (2016). The annealing temperature dependence of anatase TiO_2_ thin films prepared by the electron-beam evaporation method. Semicond. Sci. Technol..

[32] Timoumi A., Alamri S.N., Alamri H. (2018). The development of TiO_2_-graphene oxide nano composite thin films for solar cells. Results Phys..

[33] Tong W., Zhang Y., Yu L., Lv F., Liu L., Zhang Q., An Q. (2015). Amorphous TiO_2_-coated reduced graphene oxide hybrid nanostructures for polymer composites with low dielectric loss. Chem. Phys. Lett..

[34] Valeev R.G., Deev A.N., Surnin D.V., Kriventsov V.V., Karban O.V., Vetoshkin V.M., Pivovarova O.I. (2008). The structure study of amorphous nanocrystalline nanocomposite films of germanium by AFM and EXAFS methods. Surf. Interface Anal..

[35] Vallejo W., Rueda A., Díaz-Uribe C., Grande C., Quintana P. (2019). Photocatalytic activity of graphene oxide–TiO_2_ thin films sensitized by natural dyes extracted from Bactris guineensis. R. Soc. Open Sci..

[36] Yoon Y., Kye H., Yang W.S., Kang J.-W. (2020). Comparing Graphene Oxide and Reduced Graphene Oxide as Blending Materials for Polysulfone and Polyvinylidene Difluoride Membranes. Appl. Sci..

[37] Yu J., Yu J., Tang H.Y., Zhang L. (2001). Effect of surface microstructure on the photoinduced hydrophilicity of porous TiO_2_ thin films. J. Mater. Chem..

